# Home-literacy environments and language development in toddlers with Down syndrome

**DOI:** 10.3389/fpsyg.2023.1143369

**Published:** 2023-06-29

**Authors:** Madison S. Dulin, Susan J. Loveall, Laura J. Mattie

**Affiliations:** ^1^Department of Communication Sciences and Disorders, University of Mississippi, University, MS, United States; ^2^Department of Special Education and Communication Disorders, University of Nebraska–Lincoln, Lincoln, NE, United States; ^3^Department of Speech and Hearing Science, University of Illinois Urbana-Champaign, Champaign, IL, United States

**Keywords:** home-literacy environment, shared storybook reading, early language development, Down syndrome, child word learning

## Abstract

**Introduction:**

The present study aimed to (1) characterize the home-literacy environments (HLE) of toddlers with Down syndrome (DS) and (2) examine if richness of the HLE, child engagement during shared storybook reading activities, quality of a caregiver-child shared storybook reading activity, and exposure to language in the home environment predicted child receptive vocabulary concurrently (Time 1) and 6 months later (Time 2).

**Methods:**

Participants were toddlers with DS (*n* = 13 at Time 1, 11–29 months of age; *n* = 10 at Time 2) and their mothers. Mothers completed a *Home Literacy Environment Questionnaire* at Time 1, which was used to characterize the HLE and to calculate two composite variables: richness of the HLE and child engagement in shared storybook reading. Also at Time 1, the home language environment was measured using adult word count from the LENA Recorder DLP©. The LENA was also used to audio-record and capture the quality of a caregiver-child storybook reading task in the child’s home using the book *Dear Zoo*. At both time points, mothers completed the *MacArthur-Bates Communicative Development Inventories*, and the number of words understood variable was used to measure receptive vocabulary.

**Results/Discussion:**

Results indicated that toddlers with DS experience rich HLEs and interactive shared storybook reading encounters with their mothers. A multiple linear regression revealed that child engagement and the home language environment correlated with both toddlers’ concurrent and later receptive vocabularies, while the richness of the HLE and the shared storybook reading task emerged as moderate predictors of receptive vocabulary 6 months later.

## 1. Introduction

Down syndrome (DS) is a genetic condition caused by extra 21st chromosome material (Lejeune et al., 1959). Although there is great inter-individual variation, most individuals with DS have mild to severe intellectual disability and difficulties in speech and language (Chapman and Hesketh, 2000; Abbeduto et al., 2007). This includes delays in early language development such as first words (e.g., Abbeduto et al., 2007). Creating a rich home-literacy environment (HLE), such as providing regular access to books and participating in interactive caregiver-child shared storybook reading experiences, can have a large and positive impact on children’s language and literacy skills (Dickinson and Smith, 1994; Whitehurst et al., 1994; Bus et al., 1995). However, despite the well-documented difficulties with speech and language that are common in DS (e.g., Abbeduto et al., 2007), and despite the fact that DS is associated with a unique linguistic profile (e.g., Chapman and Hesketh, 2000; Fidler, 2005; Abbeduto et al., 2007), there has been little research examining if and how HLEs might impact outcomes in this population. The purpose of the present study, therefore, was to examine the HLEs of toddlers with DS and to determine if and how the HLE impacts receptive vocabulary both concurrently and 6 months later.

The HLE is defined as children’s exposure to, and the quality of, literacy-related activities in the home (DeTemple, 2001). Rich HLEs are positively related to several outcomes for young neurotypical children, including language, emergent literacy, and reading achievement (Dickinson and Smith, 1994; Whitehurst et al., 1994; Bus et al., 1995). However, richness of the HLE can be positively or negatively impacted by socioeconomic status (SES; e.g., Bus et al., 1995). For example, families with lower incomes may have less access to books and other learning materials in the home when compared to families of middle- and high-income, which in turn can negatively impact their children’s language and literacy development (e.g., Neuman, 1996). The HLE is often measured by parent-reported questionnaires examining the onset, frequency, and quality of shared storybook reading, the number of books available in the home, and the frequency of trips to the library (e.g., Boudreau, 2005; Peeters et al., 2009).

Caregiver-child shared storybook reading interactions are an important component of HLEs because they expose children to more complex syntactic forms and more novel vocabulary than spoken language alone (Sulzby, 1985; Teale and Sulzby, 1986; Whitehurst et al., 1994; Cunningham and Stanovich, 1998; Pillinger and Wood, 2014). In addition, shared storybook reading provides opportunities for caregivers to scaffold their child’s language development (Mason and Allen, 1986). Scaffolding is demonstrated by caregivers adapting, extending, clarifying, and/or paraphrasing stories based on their child’s current level of language and literacy, thus enhancing the child’s comprehension of vocabulary and story content (Altwerger et al., 1985). Other forms of scaffolding during shared storybook reading include the types of questions asked and interactive strategies that caregivers use to engage their child in the activity. For example, caregivers of young children can ask questions that require their child to point to familiar pictures in the book and/or to label something in the book, prompt their child’s physical engagement (e.g., helping the child to hold the book and/or turn the pages), and/or request that their child imitate words or sound effects (Haden et al., 1996). As children develop, caregivers can transition to using more complex strategies, such as talking with their child about the story in ways that extend beyond the text, expanding on their child’s responses, and even asking abstract questions that require their child to interpret, inference, or predict future story events (Haden et al., 1996). Although HLEs have often been measured via caregiver-reported questionnaires, research examining the quality of shared storybook reading for neurotypical children has more directly observed the importance of these interactions by recording and coding sessions to understand its impact on language development (e.g., Neuman, 1996).

Most studies on the HLEs of children with DS specifically have only described their HLEs via caregiver-reported questionnaires (Trenholm and Mirenda, 2006; Al Otaiba et al., 2009; van Bysterveldt et al., 2010; Ricci, 2011; Lusby and Heinz, 2020), and this body of literature has mixed findings regarding the richness of their HLEs. Some evidence suggests that young children with DS do have rich HLEs (Al Otaiba et al., 2009; van Bysterveldt et al., 2010; Lusby and Heinz, 2020; Burgoyne and Cain, 2022). For example, in two studies (Al Otaiba et al., 2009; Lusby and Heinz, 2020) with relatively large samples (*n*’s > 100) of young children with DS (1-to-6-year-olds), a majority of caregivers reported that they began reading to their child at an early age (i.e., 1–2 years) and that they read with their child regularly (i.e., ~55–60% reported reading with their child daily for anywhere from 6 to 30 min). Further, Al Otaiba et al. (2009) reported that approximately 60% of the families in their study had 100 or more children’s and adult-level books in the home. Most recently, Burgoyne and Cain (2022) noted in their study of eight caregivers of children with DS between 4 and 6 years that all caregivers reported reading to their child daily (*n* = 2) or even several times a day (*n* = 6) for 10 to 30 min. Notably, this research has reported on families from middle- to higher SES (Trenholm and Mirenda, 2006; Al Otaiba et al., 2009; van Bysterveldt et al., 2010; Lusby and Heinz, 2020; Burgoyne and Cain, 2022).

Other research has also reported rich HLEs in older, school-age children with DS from primarily middle- to high SES families. For example, using caregiver questionnaire data, van Bysterveldt et al. (2010) reported that both younger (*n* = 48 5-to-8-year-olds) and older school-age children with DS (*n* = 37 9-to-14-year-olds) had equal access to children’s and adult-level books in the home (on average 50–75 books). Across the combined samples, 66% of the caregivers reported that they began reading to their child by 12 months of age, and 90% reported that they read to and/or with their child, though only 48% reported that this was daily (van Bysterveldt et al., 2010).

In contrast, other questionnaire-based research has found that young children, adolescents, and adults with DS from primarily middle- to upper-middle SES families are not exposed to print-rich home environments (Trenholm and Mirenda, 2006). For example, in their study of 224 caregivers of individuals with DS between 1 and 41 years, Trenholm and Mirenda (2006) reported that approximately 80% of caregivers did not read with their child daily, often spent less than 15 min reading when they did engage in shared storybook reading and had a limited number of children’s books in the home. Further, although most caregivers reported reading the text and labeling pictures during shared storybook reading, only 20–30% of caregivers reported using other interactive reading strategies (e.g., asking questions about details in the story, predicting what would happen next, or asking why something happened in the story). However, this data was not broken up across the large age range, making it unclear if/how the results varied for younger versus older children, adolescents, and adults.

Only a few studies have included comparison groups, but results from these studies indicate that HLEs may not be as rich for young children with DS as they are for their neurotypical peers. For example, Ricci (2011), noted that when compared to neurotypical peers matched on chronological age, 3-to-6-year-olds with DS (*n* = 20) had less access to children’s and adult-level books and less frequent caregiver-child shared storybook reading activities in the home. However, a second sample of older children with DS (*n* = 17 8-to-13-year-olds) engaged in longer reading sessions than the younger group with DS and were explicitly taught alphabet knowledge, print knowledge, and word meanings while the younger group with DS was not (Ricci, 2011). In contrast, another questionnaire study by Westerveld and van Bysterveldt (2017) reported the HLEs of preschool-age children with DS (*n* = 31 3-to-5-year-olds) were richer than those of children with autism spectrum disorder (*n* = 80 3-to-5-year-olds). More than half of the caregivers (65%) from both groups reported that they began reading to their child before 1 year of age and owned at least 25 children’s books, but more caregivers of children with DS (i.e., 77.4%) reported reading books “very often” with their child than did caregivers of children with autism (i.e., 33.8%). Although informative, these studies did not include children younger than 3 years old, so it is unclear if the HLEs of toddlers with DS are similar or different.

Most questionnaires regarding the HLEs of children with DS have focused specifically on caregiver behaviors, but a few studies have included questions pertaining to the child’s engagement (van Bysterveldt et al., 2010; Westerveld and van Bysterveldt, 2017; Lusby and Heinz, 2020). Studies of early childhood and preschool-age children with DS suggest that they are highly engaged in shared storybook reading interactions with their caregivers. For example, Lusby and Heinz (2020) noted that roughly 70% of caregivers of younger children (1-to-6-year-olds) reported that their child “often/always” points to pictures in the book or turns the pages, and half reported that their child “often/always” names pictures in the story. However, only 33% of children “often/always” comment on the story or pictures in the book. Similarly, Westerveld and van Bysterveldt (2017) noted that caregivers of preschool-age children with DS (3-to-5-year-olds) reported that their child “often/usually” independently points to pictures in the book and/or talks about pictures in the book. In contrast, one study of elementary school-age children (van Bysterveldt et al., 2010) reported that only approximately 45% of the children in their study “often/usually” commented on pictures in the story, and only 10–30% “often/usually” asked questions about pictures, events, and/or characters in the story. However, these engagement behaviors were also more complex (e.g., asking questions) in comparison to those measured in the studies with younger children with DS (e.g., pointing to pictures). Thus, it is not clear if engagement during shared storybook reading decreases with age or if this pattern of results is simply a reflection of the types of engagement measured.

Moving beyond caregiver report, only a few studies have directly observed the quality of HLEs in DS (Fitzgerald et al., 1995; Barton-Husley et al., 2020; Burgoyne and Cain, 2022). Fitzgerald et al. (1995) examined HLEs by visiting the homes of three preschool-age children with DS twice over a 2-week period to complete a checklist of literacy artifacts (e.g., the number of children’s books available in the home) and to tape-record the children’s daily interactions, including caregiver-child shared storybook reading activities. These three HLEs were described as print-rich based on the number of children’s books in the home (75–100) and because all mothers engaged in storybook reading activities with their child at least one time during each visit. However, only 1 of 3 mothers were observed using interactive reading strategies (e.g., labeling pictures, asking questions, expanding on the written text) during a shared storybook reading activity. Though Fitzgerald et al. (1995) extended beyond the use of survey data to measure the richness of HLEs for children with DS, the generalizability of their results is limited by the small sample size.

The remaining two studies are the only ones, to our knowledge, that both directly observed HLEs of children with DS and examined if and how HLEs and shared storybook reading impacted language and literacy development in their samples. Burgoyne and Cain (2022) directly observed shared storybook reading interactions between eight children with DS (aged 4–6 years) and their parents by visiting the children’s homes to video record two separate mother–child storybook reading activities in the home using two different books. One book was to be read in its original form to illustrate a typical shared storybook reading interaction; the second book was modified to include embedded prompts (i.e., picture labeling, vocabulary, linking text to general knowledge, and inferencing). Parents used more extra-textual talk (i.e., asking questions, commenting, responding to their child) when they read the book with embedded prompts than during the typical shared storybook reading activity with their child. Further, children with DS showed greater participation in reading and produced significantly more words (*M* = 110.63, *SD* = 78.07) and a greater range of words (*M* = 47.13, *SD* = 25.0) when reading books with embedded prompts compared to when reading books during a typical reading session (words produced *M* = 52.50, *SD* = 35.84; different words *M* = 27.13, *SD* = 17.28; Burgoyne and Cain, 2022). Although the sample was small, these data support that more interactive reading strategies can promote language development in young children with DS.

Lastly, Barton-Husley et al. (2020) directly observed the use of interactive reading strategies between 22 mothers with children with DS (22-to-63-months old) compared to 22 mothers of neurotypical children matched on chronological age. The quality of the HLEs was examined by visiting the children’s homes once for 1 to 2 h to video record a mother–child shared storybook reading activity in the home. During shared storybook reading activities, mothers of children with DS were observed using interactive reading strategies (i.e., questions, descriptions, gestures, and labels) more frequently than did the mothers of the neurotypical peers (73% vs. 50% of recorded sessions). Further, their results indicated that mothers of children with DS who used a fewer total number of utterances during a shared storybook reading activity had children with higher receptive language raw scores as measured by the *Mullen Scales of Early Learning* (Mullen, 1995), indicating that mothers of children with DS adapt their language to meet the language and engagement needs of their child. For example, it is possible that mothers of children with DS with higher receptive language skills talked less because they were giving their child more time to talk, whereas mothers of children with DS with lower receptive language skills talked more to help scaffold language development. Though Barton-Husley et al. (2020) were the first to examine the relationship between maternal input and receptive vocabulary in toddlers and preschool-age children with DS, the results of their study are limited to a single time point. More research, particularly longitudinal research, is needed to understand the impact of HLEs and shared storybook reading on the word learning of young children with DS.

Although the benefits of rich HLEs and interactive shared storybook reading have been well documented in the neurotypical literature (e.g., Bus et al., 1995; Neuman, 1996), there is limited research examining if and how these impact language and literacy development for children with DS. There is a clear need to examine the HLEs of toddlers with DS and to determine if the richness of the HLEs impacts early language development concurrently and/or over time. Further, to fully capture the quality of shared storybook reading, it is necessary to go beyond caregiver report and include observational data to measure these interactions more directly. The purpose of the current study was to describe the HLEs of toddlers with DS, observe and document the quality of a shared storybook reading task between toddlers with DS and their mothers, and measure the relation between these variables and word learning in toddlers with DS. Specifically, the research questions for this current study were:

- How do mothers of toddlers with DS describe their HLEs using a parent questionnaire? Further, how do mothers of toddlers with DS report their child’s engagement during shared storybook reading activities using a parent questionnaire?
- What interactive strategies do mothers of toddlers with DS use during a shared storybook reading task (i.e., what is the quality of a shared storybook reading task)?
- Do differences in the richness of HLEs and quality of parent–child shared storybook reading relate to receptive language outcomes concurrently and 6 months later?

## 2. Materials and methods

### 2.1. Participants

Participants included toddlers with DS and their mothers who were part of a larger study examining early language development in DS. For the present study, participant data was used from two time points, referred to as Time 1 and Time 2, once the shared storybook reading task had been added to the protocol. Thirteen families participated in the study at Time 1. One of those participants did not complete the shared storybook reading task with their child because the battery in the LENA Recorder DLP^©^ sent to them died due to delayed participant compliance in completing the task. Data from 10 families were included for follow-up testing approximately 6 months later (*M* = 7.30 months, *SD* = 4.42 months) at Time 2. One participant did not have data at Time 2 as they had already completed and exited the overall study at this time, and two families were non-responsive at Time 2. One participant completed their follow-up testing 19 months later at Time 2 due to COVID-19, which paused the study in March 2020. Participants were 11–29 months at Time 1 (*M* = 17.92, *SD* = 5.27), and 16–48 months at Time 2 (*M* = 24.30, *SD* = 9.08). Additionally, 76.9% of the participants were white, and 69.2% were males. Mothers reported an annual family income ranging from $20,000 to > $300,000. Of the mothers in this study, 30.8% had attended some college, 23.1% were college graduates, 7.7% had postgraduate training, and 38.5% had a professional degree (e.g., MA, PhD). Participants were recruited from the Midwest and Southeast regions of the United States using emails and posting on social media to local DS organizations and early intervention service organizations. As reported by the participants’ mothers, all children had normal or corrected hearing and vision, and English was the primary language spoken in the home. Additional participant descriptive statistics are reported in Table 1.

**Table 1:** Participant descriptive statistics.

Variable	*M*	*S.D.*	Range
Chronological age, child^a^	17.92	5.27	11–29
Chronological age, mother^b^	33.85	5.86	21–46
Early learning composite^c^	62.46	10.25	49–82
Words understood^d^			
Time 1	100.15	82.67	0–301
Time 2	138.10	114.97	6–309
Words produced^d^			
Time 1	6.08	7.54	0–25
Time 2	15.10	21.02	0–66
	*f*	%	
Family income			
<$50,000	2	15	
$50,001–$100,000	6	46	
$100,001–$150,000	3	23	
$150,001–$200,000	0	0	
$200,001–$250,000	0	0	
$250,001–$300,000	0	0	
>$300,000	1	8	
Not reported	1	8	

a Child’s age is reported in months at Time 1.

b Mother’s age is reported in years at Time 1.

c Composite scores derived from *Mullen Scales of Early Learning* (MSEL) at Time 1.

d Raw scores derived from *MacArthur-Bates Communicative Development Inventories* (CDI).

### 2.2. Procedures

In-person visits were conducted at both time points in the family’s home or at a location nearby (e.g., library, DS community center). As part of a larger-assessment battery, the *MacArthur-Bates Communicative Development Inventories* (CDI) was completed by the child’s mother, and the *Mullen Scales of Early Learning* (MSEL) was administered to the child by a trained examiner. Families were also provided with a *Home Literacy Environment Questionnaire* and a LENA Recorder DLP^©^ with instructions for a shared storybook reading task using the book *Dear Zoo* (Campbell, 1982).

### 2.3. Measures

#### 2.3.1. Home-literacy environment questionnaire

For this study, we created an HLE questionnaire (adapted from Boudreau, 2005; Peeters et al., 2009; van Bysterveldt et al., 2010; 5 min; see Supplementary material) to characterize the richness of the HLE and child engagement in reading. The questionnaire included short answer (*n* = 3), Likert-type (*n* = 6), and forced choice (*n* = 19) questions. From this questionnaire, two composite variables were calculated and used in the regression analyses (research question 3): (1) parent-reported richness of the HLE and (2) parent-reported child engagement in shared storybook reading.

##### 2.3.1.1. Richness of the HLE

The composite for parent-reported richness of the HLE was comprised of 13 questions pertaining to the exposure and nature of literacy-related activities in the home, including accessibility of children’s and adult-level books in the home, parental perspectives on the importance of reading with children, amount of time spent reading, number of books read to their children, and interactive reading styles utilized during a typical shared storybook reading interaction. The sum of the 13 items was used as an indicator of the richness of the HLE, with higher scores representing richer HLEs (highest possible score: 67).

##### 2.3.1.2. Child engagement

The composite for child engagement in shared storybook reading was comprised of eight questions related to what the child does during a typical shared storybook reading interaction. The sum of the eight items was used as an indicator of child engagement in reading, with a higher score representing greater interest and engagement in shared storybook reading (highest possible score: 36).

#### 2.3.2. Language environment analysis recorder and software^©^

The LENA Recorder DLP^©^ and LENA software are a system for analyzing the language environment of a child in their day-to-day life (LENA Research Foundation, 2018). The LENA Recorder DLP^©^ is a small, wearable recorder that records for up to 16 h (when children are napping or bathing, caregivers are instructed to leave the recorder on nearby). From these recordings, adult word count, child vocalizations, and conversational turns can be extracted using the LENA PRO^©^ or SP^©^ software (LENA Research Foundation, 2018). The normative sample of the LENA software is based on audio of 2-to-48-month olds from families of varying socioeconomic backgrounds (Gilkerson et al., 2008), and norms were developed from recordings captured in a 12 h long, spontaneous speech environment (Gilkerson et al., 2008; LENA Research Foundation, 2018).

##### 2.3.2.1. Home language environment

In the present study, the home language environment was measured using the adult word count (AWC) from the LENA Recorder DLP^©^, which is automatically calculated by the LENA software.

##### 2.3.2.2. Shared storybook reading task

For the shared storybook reading task, caregivers were instructed to read the age-appropriate, lift-the-flap book, *Dear Zoo* (Campbell, 1982) during any normal reading time in a quiet environment when the child was wearing the LENA Recorder DLP^©^. The book contains predictive text and several unfinished sentences noted by an ellipsis to prompt the reader(s) to open the flap and label the pictured animal (i.e., *So they sent me a...*). Mothers were asked to note the date and time that they read the story, so that this interaction could be identified in the LENA recording for transcription. *Systematic Analysis of Language Transcription* (SALT; Miller and Iglesias, 2006) software and conventions were used to transcribe the samples and then coded using an adapted coding scheme to measure the quality of the caregiver-child shared storybook reading task, including the use of maternal interactive reading styles (i.e., Neuman, 1996; Crowe, 2000; Justice et al., 2003; McDuffie et al., 2018). Specifically, all maternal reading behaviors were coded for labels, expansions, questions, comments, requests, reading written text verbatim, paraphrasing the story, repetition of questions, and non-story speech. A composite variable was calculated to represent the quality of caregiver-child shared storybook reading for use in regression analyses (research question 3, see details below).

###### 2.3.2.2.1. Transcriber training

The primary transcriber, the first author, was trained using SALT software conventions (Miller and Iglesias, 2006). Training also included independent transcription of practice language samples from a different mother–child shared storybook task with children with DS. Each practice language sample transcript was compared to a standard transcript at the utterance level for all maternal utterances across multiple transcription dimensions. Once the primary transcriber transcribed two consecutive transcripts with at least 70% agreement for utterance segmentation, word identification, and number of morphemes and words and at least 80% agreement on the dimensions of unintelligibility, abandoned utterances, mazes, overlapping speech, and ending punctuation, they were considered trained to fidelity.

###### 2.3.2.2.2. Transcription of shared storybook reading task

Each shared storybook reading task was independently transcribed by the primary transcriber and then independently checked by another transcriber trained on SALT software conventions. Maternal utterances were segmented into Communication units (C-Units; i.e., an independent clause and any modifiers, which could include a dependent clause), recommended for individuals over 3 years (Loban, 1976). There were 87 discrepancies between the primary transcriber and checker. These were reconciled by the primary transcriber.

###### 2.3.2.2.3. Coding of shared storybook reading task and outcome variables

The quality of the shared storybook reading task was measured via an adapted coding scheme (i.e., Neuman, 1996; Crowe, 2000; Justice et al., 2003; McDuffie et al., 2018). This adapted coding scheme focused on the use of maternal reading behaviors (i.e., labels, expansions, questions, comments, requests, reading written text verbatim, paraphrasing the story, repetition of questions, and non-story speech). All maternal reading behaviors during the shared storybook reading task were coded and summed to create a total maternal utterances variable. Maternal utterances that were coded as labels, expansions, questions, comments, and requests were considered “interactive” reading strategies. Reading the written text verbatim, paraphrasing the text, repetition of questions, and non-story speech were considered codable utterances but were not counted as interactive reading strategies. Coded maternal reading behaviors and examples of each can be found in Table 2.

**Table 2:** Maternal reading behaviors during the caregiver-child shared storybook reading task.

Code	Definition	Example
*Labels	Label of a pictured agent or object following a spontaneous question (i.e., a question not included in the text) that typically elicited a one-word naming response	Mother: “What is it?” “Giraffe!”
*Expansions		
Comment	Added information that was outside the written text and/or comments on the pictures	Mother: “The giraffe is in the box.”
Bridge	Making connections from story content to everyday experiences	Mother: “We went to the zoo and saw a tall giraffe.”
*Questions		
Yes/No	Question that required a “yes” or “no” response from the child	Mother: “Do you see the giraffe?”
Tag	Question that could be answered by “yes” or “no” but typically was rhetorical or served as a form of commenting	Mother: “He’s in a box, is not he?”
Label	Question that typically elicited a one-word naming response	Mother: “What is that?”
Descriptive	Question that required a response to describe an action related to the story	Mother: “What is happening in this picture?”
Complex	Question that required a semantically complex response (i.e., predictive, casual)	Mother: “What will happen next?”
Choice	Question that required a choice from the child	Mother: “Is the giraffe big or little?”
*Comments	General comment that related to the shared storybook reading activity	Mother: “I really like this story.”
*Requests		
Imitative	Request for imitation	Mother: “Say, ‘giraffe’!”
Sound effects	Request for sound effect	Mother: “What does a lion say?”
Reading text verbatim	Utterance that was verbatim from the written text	Mother (reading text): “I wrote to the zoo to send me a pet.”
Text completion	Label (i.e., one-word naming response) of a pictured agent or object that completed the written text, “*So they sent me a(n)…*”	Mother (reading text): “So they sent me a…” Mother (opens flap to see picture): “Giraffe!”
Paraphrasing	Utterance that paraphrased the story but was not reading the written text verbatim	Text: “They sent me an…” Paraphrase: “And here’s what they sent me.”
Repetition of questions	Utterance that repeated child’s utterance as a question	Child: “Giraffe.” Mother: “Giraffe?”
Non-story speech	An utterance that did not contain story content or did not relate to the reading task (e.g., side conversation or behavior management: attention, non-desirable, praise)	Mother: “Stop doing that.”

*Denotes that these codes were considered “interactive” reading strategies when calculating the SBR composite score measuring the quality of the caregiver-child shared storybook reading task for use in regression analyses; research question 3.

Three primary outcome variables were calculated from the shared storybook reading task. First, the number of mothers who used each interactive strategy were counted. Second, the percentage of each of the interactive reading strategies used by the mothers was calculated (i.e., percentage of total maternal utterances that were labels, expansions, questions, comments, or requests). Third, a shared storybook reading (SBR) composite score was calculated to represent the quality of the task for use in regression analyses. An SBR was calculated for each participant by dividing their total number of utterances that were interactive strategies by their total number of maternal utterances. Then, the SBR composite was calculated by averaging individual SBR scores across participants.

###### 2.3.2.2.4. Coder training

The primary transcriber also served as the primary coder. First, the primary coder discussed the adapted coding scheme with a second, reliability coder by providing definitions and examples of each code. The primary coder then shared a coded transcript with the reliability coder and discussed each coding choice.

###### 2.3.2.2.5. Coding reliability

After coder training, each shared storybook reading task was independently coded by the primary coder and independently reviewed by the reliability coder. There were 21 discrepancies in coding. These were reconciled by the primary coder.

#### 2.3.3. Vocabulary

The *MacArthur-Bates Communicative Development Inventories-Words & Gestures* (CDI; Fenson et al., 2007) was used to measure the children’s receptive vocabulary (i.e., words understood) at both Time 1 and Time 2. The CDI is a standardized parent-reported checklist designed to assess children’s receptive and expressive vocabulary skills from 8 to 30 months. The CDI has good reliability and validity (Fenson et al., 2007). The internal consistency coefficients for the vocabulary scales range from 0.95 to 0.96, and test–retest reliability coefficients for Words and Gestures range from 0.61 to the mid 0.80s. The CDI Words and Gestures correlates (i.e., has concurrent validity) with the *Expressive One-Word Picture Vocabulary Test* (Gardner, 1981), with correlations ranging from 0.73 to 0.86 and with the *Reynell Developmental Language Scales*, *Expressive* (Reynell and Gruber, 1990), with correlations ranging from 0.75 to 0.82. The CDI has previously been used with children with DS, and moderate to strong correlations have been reported between the CDI and other measures of vocabulary (0.70; Miller et al., 1995) and language (0.77; i.e., *Bayley Scales of Infant Development*; Heilmann et al., 2005) for children with DS. Words understood was used as the outcome variable. Words produced was not used in this study because very few participants were using spoken language at this time.

#### 2.3.4. Cognitive abilities

The *Mullen Scales of Early Learning* (MSEL; Mullen, 1995) was administered by a trained examiner to measure the children’s overall cognitive abilities at Time 1. The MSEL is a standardized assessment tool designed to measure development from birth to 68 months of age across four domains, Visual Reception, Fine Motor, Receptive Language, and Expressive Language, which together yield an overall measure of cognitive abilities, the Early Learning Composite (ELC). Like other standardized, norm-referenced assessments of cognition (used to estimate level of intellectual abilities), the ELC has a mean of 100 and a standard deviation of 15. In addition, an overall developmental age can be calculated by averaging the age equivalents from these four domains. The internal consistency coefficients range from 0.83 to 0.95, test–retest reliability coefficients range from 0.82 to 0.85, and interrater reliability coefficients range from 0.91 to 0.99 (Mullen, 1995). The MSEL has strong concurrent validity with other standardized tests of early child development and cognition [e.g., *Bayley Scales of Infant Development* (Bayley, 1993), *Peabody Developmental Motor Scales* (Folio and Fewell, 1983), *Birth to Three Developmental Scale* (Dodson and Bangs, 1979)]. Additionally, content validity and construct validity have been established (Mullen, 1995).

### 2.4. Data analysis plan

For research question 1, descriptive statistics were used to report how mothers of toddlers with DS characterize their HLEs, use of interactive reading styles, and child engagement via the HLE questionnaire. For research question 2, descriptive statistics were again used to describe the quality of a shared storybook reading activity between mothers and their child with DS via coding of a caregiver-child shared storybook reading task. For research question 3, multiple linear regression was used to examine if the richness of parent-reported HLEs, parent-reported child engagement in reading activities, the quality of a caregiver-child shared storybook reading task, and the home language environment predicted receptive language outcomes at Time 1 and Time 2. Words understood at Time 1 was slightly skewed (Skewness = 1.12), and several variables had slightly elevated kurtosis values (words understood Time 1 = 1.55, words understood Time 2 = −1.50, child engagement = −1.17, adult word count = −1.20). For the regression predicting words understood at Time 1, there were no serious violations of the assumptions of multiple regression, including multicollinearity. For the regression predicting words understood at Time 2, there were two participants identified as having standardized residuals above 3.0 or below −3.0. These were the participants with the highest and lowest words understood scores, 6 (this was the youngest participant) and 158, respectively. Because the sample size was already reduced at Time 2, and because we believed these scores reflected each participant’s true receptive vocabulary abilities, we elected to keep these participants in the analyses. There were no other major violations of multiple regression, including multicollinearity. Follow-up analyses were also run adding age to the regression models as a control variable. Given the small sample size and that we already had four predictors (i.e., our primary predictors of interest: HLE, caregiver-child shared storybook reading, child engagement in shared storybook reading, and the home language environment), we added age as a predictor after running the originally proposed regressions. Further, because participants had younger developmental ages, we consider the regressions with age exploratory and suggest caution in interpreting these findings. Further, given that one participant was not able to complete the Time 2 session until 19 months later (see participant information above), the Time 2 regression analyses were run both with and without this participant included. The pattern of results did not change, and the statistical results themselves changed only minimally. Thus we retained this participant in our presented results. Descriptive statistics of key variables can be found in Table 3. Correlations among key variables can be found in Table 4.

**Table 3:** Descriptive statistics of key variables.

Variable	*M*	*S.D.*	Range	*N*
HLE^a^	34.89	8.12	21.0–49.0	13
Child engagement^b^	12.38	6.38	4.0–23.0	13
Home language environment (AWC)^c^	17,142.62	8,867.64	4,865–30,675	13
SBR^d^	0.35	0.12	0.20–0.60	12

a Composite score derived from the HLE questionnaire to describe the richness of the HLE.

b Composite score derived from HLE questionnaire to describe child engagement during typical shared storybook reading activities.

c Adult word count (AWC) derived from LENA Recorder DLP^©^ to measure home language environment.

d Composite score representing the average quality of the shared storybook reading task across participants.

**Table 4:** Correlations among key variables.

Variable	1	2	3	4	5	6	7	8
1. Child age	–							
2. HLE	0.42	–						
3. Child engagement	0.77**	0.32	–					
4. Home language environment (AWC)	−0.19	−0.21	0.07	–				
5. SBR	0.07	−0.08	0.11	0.18	–			
6. WU Time 1	0.83***	0.41	0.82***	−0.40	−0.08	–		
7. WU Time 2	0.79**	0.52	0.89***	−0.23	0.29	0.89***	–	
8. ELC	−0.37	−0.54^+^	0.05	0.25	0.16	−0.27	−0.08	–

HLE, Richness of HLE; AWC, Adult Word Count; SBR, Quality of Shared-Book Reading Task; WU Time 1, Words Understood at Time 1; WU Time 2, Words Understood at Time 2; ELC, Early Learning Composite derived from *Mullen Scales of Early Learning* (MSEL).

^+^*p* < 0.07, **p < 0.01, ***p < 0.001.

## 3. Results

### 3.1. Research question 1: home-literacy environment, Time 1

On the HLE questionnaire, all mothers reported that they began reading to their child between pregnancy and 12 months of age (*M* = 1.88, *SD* = 4.05), and approximately half (*n* = 6) had a designated reading time with their child. Mothers also reported that they had many children’s (*M* = 122.31, *SD* = 99.26, *range* = 30–300) and adult-level books (*M* = 120.77, *SD* = 74.77, *range* = 20–200) in their home. Most mothers (*n* = 11) reported reading with their child regularly, and when asked about different interactive reading strategies during caregiver-child shared storybook reading, a majority agreed (i.e., “strongly agree”/ “agree”) that they use interactive strategies during typical storybook reading activities with their child (*n* = 13 point out details outside the written text; *n* = 10 relate the story to the child’s experiences; *n* = 10 teach their child letters and sounds). The exception was that only half (*n* = 7) agreed that they ask their child questions about the story and follow-up with answers. Frequency of mother responses to additional items on the HLE questionnaire can be found in Table 5.

**Table 5:** Frequency of mothers’ responses to items on HLE questionnaire related to the richness of HLE.

Question	Never/0	1–2	3–4	5–6	7–8	9–10	11+
How many times in a week mother reads books for enjoyment	3	4	4	0	1	1	0
How many times in a week mother reads informative books	3	7	1	2	0	0	0
How many times in a week mother reads to child	0	0	2	3	2	3	3
How many books mother read to child in past week	0	1	0	4	2	1	5
How many books mother reads to child in one sitting	0	5	7	1	0	0	0
							
	**<15 min**	**15–30 min**	**30–45 min**	**1–2 h**	**3–4 h**	**5–6 h**	**7+ h**
How much time mother spent reading to child in past week^a^	0	0	4	3	4	1	0
							
	**<10 min**	**10–20 min**	**21–30 min**	**31–40 min**	**41–50 min**	**51–60 min**	**>1 h**
How much time mother spends reading to child in one sitting	2	8	2	1	0	0	0
							
	**Never**	**Once**	**Every 4–6 mos**	**Every 2–3 mos**	**Monthly**	**Every 2–3 wks**	**Weekly**
How often mother took child to library/bookstore in the last year	1	3	2	0	0	4	3

Interactive book reading styles	Strongly disagree	Disagree	Agree	Strongly agree
Points out details outside of written text	0	0	6	7
Relates the story to child’s everyday experiences	0	3	4	6
Asks questions and follows-up with answers	2	4	7	0
Teaches child letters/sounds of letters	1	2	7	3

*N* = 13. Min, Minutes; H(s), Hour(s); Wks, Weeks; Mos, Months.

^a^One participant did not report an answer.

When asked about their child’s engagement in shared storybook reading activities, approximately half (*n* = 6) reported that their child asks to read books or pretends to read the story in a book (e.g., sitting with a book and producing speech that is similar to the actual story in the book). When asked questions pertaining to the child’s engagement during book reading, most mothers reported that their child interacts with the story by grabbing for and/or holding the book (*n* = 13), turning the pages (*n* = 12), and pointing to pictures or words on the page (*n* = 8). In contrast, most mothers reported that their child does not regularly name familiar pictures in the book (*n* = 12), ask questions about the story (*n* = 13), or fill in words or lines of a familiar story (*n* = 13). Mothers’ responses to items on the HLE questionnaire related to child engagement in reading can be found in Table 6.

**Table 6:** Frequency of mothers’ responses to items on HLE questionnaire related to child engagement.

Question	Never	1–2	3–4	5–6	7–8	9–10	11+
How many times child asks mother to read to him/her in a week	7	2	1	1	0	0	2
How many times child pretends to read books in a week^a^	6	1	0	1	2	0	2

“During storybook reading time with your child, does he/she…”	Never	Has but rarely	Occasionally	A few times per story	Frequently during story
Grabs for/holds book	0	0	3	1	9
Turns pages of book	1	0	2	1	9
Points to pictures/words in book independently	5	0	3	2	3
Names familiar pictures	9	3	0	1	0
Asks questions about story	13	0	0	0	0
Fills in words/lines of a familiar story	13	0	0	0	0

*N* = 13.

^a^One participant did not report an answer.

### 3.2. Research question 2: shared storybook reading task, Time 1

Frequency counts of the number of mothers who used each interactive shared storybook reading strategy are reported below. Means, standard deviations, ranges, and percentages of all maternal coded reading behaviors at Time 1 are presented in Table 7. Expansions, questions, and labels made up the majority of the interactive reading strategies used. Requests and comments were used less often.

**Table 7:** Use of interactive reading strategies by type during the caregiver-child shared storybook reading task.

Variable	Total number	*M*	*S.D.*	Range
Maternal utterances	801	66.75	23.14	43–116
Utterances that were interactive strategies	297	24.75	16.97	9–70
	Total number (% of maternal utterances)	*M*	*S.D.*	Range
*Labels	69 (8.61)	5.75	5.71	0–21
*Expansions				
Comment	87 (10.86)	7.25	5.26	2–19
Bridge	13 (1.62)	1.08	1.08	0–3
*Questions				
Yes/No	17 (2.12)	1.42	1.68	0–5
Tag	1 (0.12)	0.08	0.29	0–1
Label	65 (8.11)	5.42	5.55	0–19
Descriptive	4 (0.50)	0.33	0.89	0–3
Complex	3 (0.37)	0.25	0.62	0–2
Choice	1 (0.12)	0.08	0.29	0–1
*Comments	17 (2.12)	1.42	1.56	0–5
*Requests				
Imitative	14 (1.75)	1.17	2.59	0–8
Sound effects	6 (0.75)	0.5	1.17	0–4
Reading text verbatim	323 (40.32)	26.92	4.19	21–37
Text completion	47 (5.87)	3.92	2.78	0–8
Paraphrasing	15 (1.87)	1.25	1.91	0–6
Repetition of questions	9 (1.12)	0.75	1.06	0–3
Non-story speech	110 (13.73)	9.17	6.94	1–28

*N* = 12.

* Were considered “interactive” reading strategies when calculating the composite score measuring the quality of the caregiver-child shared storybook reading task for use in regression analyses; research question 3.

#### 3.2.1. Labels

Ten of the 12 mothers used labels by asking spontaneous questions (e.g., “What is that?) that they then followed with a one-word naming response (e.g., “Giraffe!”).

#### 3.2.2. Expansions

All 12 mothers expanded on the written text and/or commented on the pictures during the shared storybook reading task. Seven of the 12 mothers used expansion-bridges by connecting the story content to the child’s everyday experiences during the shared storybook reading task.

#### 3.2.3. Questions

Seven of the 12 mothers asked yes-or-no questions during the shared storybook reading task. Ten of the 12 mothers asked labeling questions during the shared storybook reading task. Two mothers asked descriptive questions, and two mothers asked complex questions during the shared storybook reading task. Only one mother asked a tag and/or a choice question during the shared storybook reading task.

#### 3.2.4. Comments

Eight of the 12 mothers made general comments related to the shared storybook reading task.

#### 3.2.5. Requests

Three of the 12 mothers requested that their child imitate a word during the shared storybook reading task. Similarly, three mothers requested that their child make sound effects during the shared storybook reading task.

### 3.3. Research question 3: predicting child word learning

#### 3.3.1. Time 1

Multiple linear regression was conducted with parent-reported richness of the HLE (via the HLE composite from the HLE questionnaire), parent-reported child engagement in book reading (via the child engagement composite from the HLE questionnaire), quality of the shared storybook reading task (via the SBR composite from the LENA), and the home language environment (via AWC from the LENA) as predictors of child receptive vocabulary at Time 1. A significant model emerged, *F*(4, 7) = 15.10, *p* = 0.001, with approximately 90% of the variance in child receptive vocabulary accounted for by the linear combination of predictors (see Table 8). Examination of the predictor variables showed significant, unique effects, with child engagement explaining 63% unique variance and home language environment (AWC) explaining 17% unique variance. See the Supplementary materials for scatterplots of the data.

**Table 8:** Multiple linear regression predicting receptive vocabulary at Time 1.

Predictor variable	Beta	*t*	*p*
HLE	0.04	0.27	0.79
Child engagement	0.85	6.51	<0.001
SBR	−0.10	−0.76	0.47
Home language environment (AWC)	−0.44	−3.42	0.01

*N* = 12. HLE, Richness of HLE; SBR, Quality of Shared-Book Reading Task; AWC, Adult Word Count.

##### 3.3.1.1. Age

A follow-up analysis was conducted to examine the role of age in our regression model. Thus, we performed the analysis again but added age as a control variable. When age from Time 1 was entered alone, it significantly predicted child receptive language at Time 1, *F*(1, 10) = 21.84, *p* < 0.001, and the *R*^*2*^ indicated that 69% of the variance in child receptive language was accounted for by age (see Table 9). When the other variables were added to the model, the model remained significant, *F*(5, 6) = 13.31, *p* = 0.003, with approximately 92% of the variance in child receptive vocabulary accounted for by the linear combination of predictors. This accounted for an additional 23% of the variance in child receptive language, *F change* (4, 6) = 4.20, *p* = 0.06. Examination of the predictor variables in Step 2 indicated that age was no longer a significant predictor, but child engagement explained 16% unique variance and home language environment (AWC) explained 12% unique variance.

**Table 9:** Multiple linear regression predicting receptive vocabulary at Time 1 accounting for age at Time 1.

Predictor variable	*B*	Beta	*t*	*p*
Model 1				
Age	13.14	0.83	4.67	<0.001
Model 2				
Age	4.06	0.26	1.27	0.25
HLE	0.06	0.01	0.05	0.97
Child engagement	8.74	0.65	3.40	0.01
SBR	−74.47	−0.11	−0.87	0.42
Home language environment (AWC)	−0.003	−0.38	−2.90	0.03

*R*^2^ = 0.66 for Model 1; *R*^2^ change = 0.23 for Model 2; Total *R*^2^ = 0.92. HLE, Richness of HLE; SBR, Quality of Shared-Book Reading Task; AWC, Adult Word Count.

#### 3.3.2. Time 2

Multiple linear regression was conducted to examine if Time 1 measures (parent-reported richness of the HLE, parent-reported child engagement in book reading, quality of the shared storybook reading task, and the home language environment) predicted child receptive vocabulary at Time 2. A significant model emerged, *F*(4, 5) = 119.80, *p* = < 0.001, with approximately, 99% of the variance in child receptive vocabulary at Time 2 accounted for by the linear combination of predictors (see Table 10). Examination of the predictor variables showed a significant, unique effect for all predictors: richness of the HLE (4% unique variance explained), child engagement (57%), quality of the shared storybook reading task (SBR; 7%), and home language environment (AWC; 8%). See the Supplementary materials for scatterplots of the data.

**Table 10:** Multiple linear regression predicting receptive vocabulary at Time 2.

Predictor variable	Beta	*t*	*p*
HLE	0.23	4.53	0.006
Child engagement	0.81	16.67	<0.001
SBR	0.27	5.86	0.002
Home language environment (AWC)	−0.29	−6.10	0.002

*N* = 10. HLE, Richness of HLE; SBR, Quality of Shared-Book Reading Task; AWC, Adult Word Count.

##### 3.3.2.1. Age

Again, we conducted a follow-up analysis to examine the role of age, with age added to our regression model as a control variable. When age at Time 1 was entered alone, it significantly predicted child receptive language at Time 2, *F*(1, 8) = 13.16, *p* = 0.007, and the *R^2^* indicated that 62% of the variance in child receptive language at Time 2 was accounted for by age at Time 1 (see Table 11). When the other Time 1 measures were added to the model, the model remained significant, *F*(5, 4) = 12.06, *p* = 0.016, with approximately 94% of the variance in child receptive vocabulary accounted for by the linear combination of predictors. This accounted for an additional 32% of the variance in child receptive language, *F change* (4, 4) = 5.08, *p* = 0.07. Examination of the predictor variables in Step 2 indicated that age no longer contributed unique significant effects. Further, richness of the HLE, quality of a shared storybook reading task, and the home language environment (AWC) did contribute to the overall model when age was *not* in the model (see Table 10), this was no longer the case once age was entered into the model. Nonetheless, child engagement remained significant, explaining 22% of the variance when age was in the model.

**Table 11:** Multiple linear regression predicting receptive vocabulary at Time 2 accounting for age at Time 1.

Predictor variable	*B*	Beta	*t*	*p*
Model 1				
Age Time 1	16.20	0.79	3.63	0.007
Model 2				
Age Time 1	−2.69	−0.13	−0.51	0.64
HLE	3.09	0.24	1.77	0.15
Child engagement	15.20	0.93	3.72	0.02
SBR	121.62	0.10	0.79	0.48
Home language environment (AWC)	−0.003	−0.26	−1.73	0.16

*R*^2^ = 0.62 for Model 1; *R*^2^ change = 0.32 for Model 2; Total *R*^2^ = 0.94. HLE, Richness of HLE; SBR, Quality of Shared-Book Reading Task; AWC, Adult Word Count.

## 4. Discussion

The purpose of the current study was to examine the HLEs of toddlers with DS and determine if and how the HLE impacts their early word learning. Specifically, we collected data to describe the HLEs of toddlers with DS, documented the quality of a shared storybook reading task between those toddlers and their mothers, and measured the relationship between these variables and early language development. Although HLEs have been documented as important predictors of language and literacy outcomes in neurotypical children (e.g., Dickinson and Smith, 1994; Whitehurst et al., 1994; Bus et al., 1995), there has been limited research on the HLEs of children with DS. Further, our study added to the existing DS literature by examining child engagement during caregiver-child shared storybook reading via the use of an HLE questionnaire, directly measuring the quality of a shared storybook reading task via the use of a LENA recorded language sample, and including a longitudinal component to examine early language in toddlers with DS. Overall, our results indicate that some toddlers with DS, at least those from relatively higher socioeconomic status families, have rich HLEs and that child engagement and the home language environment correlate with their concurrent and later receptive vocabulary abilities. Richness of the HLE and quality of the shared storybook reading task also emerged as moderate predictors of word learning 6 months later. Interestingly, while age alone correlated with both concurrent and later child receptive vocabulary, once variables related to HLE and shared storybook reading were added to regression models, age was no longer a significant correlate. Below we review our findings in more detail and discuss their implications for caregivers and practitioners.

### 4.1. Richness of the HLE

Our first research question was how mothers of toddlers with DS characterize their HLEs and book reading styles. The caregivers in our study indicated that on average there were 100 children’s books in the home. Further, more than half of the mothers reported that they read with their child 7–11 times a week and spent 10–30 min reading together per session. These findings are consistent with previous research suggesting rich HLEs for young children with DS (Al Otaiba et al., 2009; van Bysterveldt et al., 2010; Lusby and Heinz, 2020; Burgoyne and Cain, 2022). For example, Al Otaiba et al.’s (2009) findings suggest that young children with DS (aged 1–6 years) have access to 100–200 children’s books in the home and are read to daily for 10–30 min. Our findings also extended Al Otaiba et al.’s work by suggesting that mothers begin establishing these patterns when their children are toddlers. Thus, it seems that toddlers with DS, at least those in our study who came from relatively high socioeconomic backgrounds (e.g., almost all mothers had some college education), have rich HLEs that include access to books and regular shared storybook reading experiences with their caregivers.

Additionally, most mothers in our study reported using interactive reading styles (i.e., point out details from the story that are outside the written text, relate the story’s content to their child’s everyday experiences, teach alphabet letters and/or sounds, ask their child questions about the story and follow-up with answers) during shared book reading with their child. Our results are consistent with Barton-Husley et al. (2020) who reported that mothers of children with DS asked more questions and used more descriptions, gestures, and labels during a caregiver-child shared storybook reading activity than mothers of neurotypical children. Although the current study relied on a parent-reported questionnaire to characterize book reading styles and did not include a comparison group, our findings indicate that mothers of toddlers with DS are using interactive strategies during shared storybook reading.

Lastly, we asked caregivers to report on their child’s engagement during shared storybook reading activities. Half of the mothers reported that their child asks them to read to him/her or pretends to read a book on their own during a typical week. Most mothers reported that during typical shared storybook reading activities, their child “very frequently” grabs for and/or holds the book and turns the pages. However, most mothers reported that their child was less likely to engage during the book reading activity with more advanced engagement behaviors (i.e., naming familiar pictures, asking questions about the story, or filling in words/lines of a familiar story). Our questions on child engagement were adapted from Peeters et al. (2009) who examined early patterns of child engagement (e.g., grabbing for or holding the book, turning pages of the book, pointing to pictures or words on the page) in slightly older neurotypical children who were on average 72 months old. Our pattern of results likely reflects our participants’ younger ages (11–29 months) and are consistent with Barton-Husley et al. (2020), who reported that during shared storybook reading activities, toddlers and preschool-age children with DS used more gestures (e.g., pointing to pictures, head nods) and vocalizations (i.e., intentional communicative sounds) than their neurotypical peers but fewer verbalizations (i.e., words, word approximations). Additionally, Lusby and Heinz (2020), noted that when caregivers of 1-to-6-year-old children with DS were asked about child engagement during shared storybook reading activities via a caregiver-reported questionnaire, roughly 70% of children were reported to “often/always” point to pictures in the book or turn the pages during shared storybook reading activities.

### 4.2. Quality of a shared storybook reading task

Our second research question was to assess the quality of a shared storybook reading activity between toddlers with DS and their mothers. Our results suggest that mothers of toddlers with DS used a high percentage of expansion-comments, labels, and labeling questions during shared storybook reading. They used a moderate amount of expansion-bridges, yes-or-no questions, comments, and requests. In contrast, they used a low percentage of tag, descriptive, complex, and choice questions. This pattern of results appears consistent with previous research examining young children with DS that suggests mothers adapt their language to meet their child’s developmental level (e.g., Barton-Husley et al., 2020; Burgoyne and Cain, 2022). For example, Barton-Husley et al. (2020) reported that mothers of children with DS used more questions, descriptions, gestures, and labels (i.e., interactive reading strategies) during a caregiver-child shared storybook reading activity when compared to mothers of neurotypical children who simply read the text verbatim. As children with DS get older and develop stronger language skills, mothers may begin using more complex reading strategies. This would be consistent with previous research on older neurotypical children, in which mothers used more complex reading strategies such as talking with their child about the story in ways that extend beyond the written text, expanding on their child’s utterances, and asking complex, descriptive, and/or abstract questions about the story events (Altwerger et al., 1985; Haden et al., 1996).

### 4.3. Predicting child word learning

Our third research question asked if the richness of HLEs and the quality of caregiver-child shared storybook reading, as well as child engagement in shared storybook reading and the home language environment, related to receptive vocabulary concurrently and 6 months later. Child engagement in shared storybook reading activities emerged as the strongest, unique predictor of child receptive vocabulary concurrently and 6 months later; children who were reported as being more engaged had larger receptive vocabularies at both time points. This relation may be explained in one of three ways. First, children who have stronger receptive vocabularies may be more engaged in book reading (e.g., van der Schuit et al., 2009). Second, children who are more engaged in book reading may develop stronger receptive vocabularies because of actively participating in shared storybook reading activities. Third, there is a transactional relationship occurring between child engagement and child receptive word learning, in which children with stronger receptive language are more likely to engage in book reading activities with their caregivers. Then, as a result of spending more time with their caregiver in shared storybook reading, these children continue to develop stronger receptive vocabularies (Sameroff, 1975; Mattie and Hadley, 2021).

The home language environment, as measured by adult word count, was also a significant, unique predictor of child receptive vocabulary concurrently and 6 months later. Interestingly though, these variables were inversely related: the larger the adult word count, the smaller the child’s receptive vocabulary. Barton-Husley et al. (2020) found a similar pattern when examining maternal input and child language comprehension during shared storybook reading activities in young children with DS. Those mothers of children with DS were found to use fewer total number of utterances when their child’s receptive language skills were higher (Barton-Husley et al., 2020). Similarly, in the current study, caregivers of toddlers with less language talked more to their child. This may be a reflection of caregivers adapting their language to the needs/abilities of their child to help them learn new words and communicate. For example, caregivers of toddlers with higher receptive language skills may talk less because they are giving their child more time to talk (Mattie and Hadley, 2021) and/or more time to process what is being said to them. This is consistent with neurotypical literature showing that caregivers alter the amount and nature of the literacy experiences they provide based on the abilities of their child (e.g., Senechal and LeFevre, 2014).

In contrast to child engagement and the home language environment, the HLE did not emerge as a significant predictor/correlate of the child’s receptive vocabulary at Time 1. Although previous research indicates rich HLEs are important to language development (e.g., Bus et al., 1995), this effect may not have been strong enough to observe in our data at Time 1, particularly in combination with our very young children with DS who were still in the early stages of language learning. At these early stages, the children’s language may just not have been developed enough to capture the importance of the HLE on receptive vocabulary. However, with ongoing exposure to print-rich environments and its cumulative impact over time, in addition to the cognitive and language development of the children, the HLE did emerge as a significant predictor at Time 2. This is consistent with previous research suggesting that HLEs are associated with vocabulary and account for approximately 40% of the variance in vocabulary growth of preschool-age neurotypical children (Storch and Whitehurst, 2001).

Quality of the shared storybook reading task also did not emerge as a significant predictor/correlate of the child’s receptive vocabulary at Time 1. However, it was a significant predictor of child word learning at Time 2. Similar to our findings for the contribution of the HLE on child word learning, it is possible that this effect may not have been strong enough to observe at Time 1 given our young participants and their early developmental levels. However, this effect became stronger at Time 2, perhaps because of the child’s increased exposure to shared storybook reading activities over time and their increasing developmental level. This finding at Time 2 is consistent with previous research suggesting that caregiver-child shared storybook reading provides opportunities for caregivers of neurotypical children and preschool-age children with DS to scaffold their child’s language based on their current language and literacy skills, therefore, enhancing their child’s comprehension (Altwerger et al., 1985; Mason and Allen, 1986; Barton-Husley et al., 2020).

Two additional regressions, one predicting word learning at Time 1 and one predicting word learning at Time 2, were run with age entered as a control variable in step 1. The overall pattern of results did not change for word learning at Time 1: when age was entered alone, it significantly predicted child receptive language, but when the other variables were added to the model, child engagement and the home language environment (AWC) were the only significant predictors. The pattern of results changed slightly when predicting word learning at Time 2: when age from Time 1 was entered alone, it significantly predicted child receptive language. However, when the other Time 1 measures were added to the model, the only significant predictor was child engagement. Age, HLE, shared storybook reading (SBR), and the home language environment were no longer significant. Thus, it seems that while chronological age may be important to consider, it is not as predictive or supportive of receptive vocabulary for young children with DS as other variables, particularly child engagement and the home language environment.

### 4.4. Limitations and future directions

There are several limitations of this study worth noting. First, our study relied solely on a parent-reported questionnaire to measure the richness of the HLE and child engagement in shared storybook reading. This may provide less reliable and/or valid data than direct assessments/observational data, especially because participants may have responded in socially desirable ways. For example, on the HLE questionnaire, caregivers may have exaggerated their estimates of the richness of the HLE and child engagement in shared storybook reading and provided biased information, even if unintentionally. Future research could develop and work to standardize and validate measures of the HLE. It is also possible that only parents who foster rich HLEs were interested in participating in the study. However, parents of neurotypical children have been found to be accurate reporters of the HLE (Boudreau, 2005). Regardless, future research should examine the impact of child engagement in shared storybook reading on language learning in DS by directly observing the child’s HLE, including caregiver-child shared storybook reading activities in the home similarly to Barton-Husley et al. (2020), who visited the participants’ homes for a single 1 to 2 h time segment and video-recorded a mother–child shared storybook reading dyad to examine maternal input and its impact on receptive language outcomes. In addition, future studies should examine the impact of the HLE and child engagement on vocabulary using applied longitudinal data analysis to observe change in vocabulary growth over time.

Second, the limited number of participants in our study (*n* = 13) makes it difficult to generalize results to the larger population of children with DS. We also had more male than female participants. Further, our sample had limited diversity, including across race and ethnicity, and we did not measure if children were exposed to additional languages. Given our small sample size, we did not include socioeconomic status in our analyses, but it is important to note that most of our participants came from families with higher socioeconomic statuses (e.g., college educated mothers, most making more than $50,000 per year). Future research with larger and more diverse samples that also considers the impact of socioeconomic status is warranted. However, this study is strengthened by its use of a longitudinal design with a 6-month interval and the participant’s narrow age range (11–29 months). Additionally, while the sample size was limited to 13 participants at Time 1 and 10 participants at Time 2, we found significant effects in our study, strengthening our confidence in the findings.

Third, our study did not include any comparison groups, making it difficult to know if/how our results compare to other populations, including neurotypical toddlers or toddlers with other intellectual and/or developmental disabilities. Therefore, future research should examine how HLEs of toddlers with DS compare to those of other children.

Fourth, in our study, a LENA Recorder DLP^©^ was used to measure the quality of a caregiver-child shared storybook reading task which solely captured the audio of speakers. Therefore, we were unable to observe non-verbal behaviors of toddlers with DS which may not fully represent the child’s communicative behaviors. Future research should use video recording to capture the non-verbal behaviors of children with DS and their parents during a caregiver-child shared storybook reading activity. Additionally, due to limitations with the LENA Recorder DLP^©^ software, we were only able to measure adult word count for the entire recording period (up to 16 h while the child was wearing the device) and not the shared storybook reading activity in isolation. In addition, the LENA Recorder DLP^©^ does not differentiate speakers, only if the speaker is an adult or child. Therefore, adult word count contains language input from any adult that is close to the child. Future research could also use audio and video recordings to code for caregiver-child interactions in the home to measure the home language environment more precisely (e.g., child-directed speech vs. total language exposure in the home). Future research should also examine the quality and complexity of caregivers’ language, for example to see if it changes over time and how this relates to and/or is impacted by the child’s speech and language development and to measure if there are differences in the language used by parents who talk more versus less (e.g., number of labels, questions, or comments used).

Finally, while our study, along with previous research in the field, relied solely on the role of mothers on their child’s language and literacy development, future research should examine other caregivers’ input on children’s early development, including, for example, fathers and grandparents. Future research should also consider the impact of having multiple caregivers contributing to HLEs and shared storybook reading experiences.

### 4.5. Implications and application

Early childhood educators, interventionists, and other practitioners play a crucial role in educating caregivers of toddlers with DS on how to promote positive language and literacy outcomes (Fidler, 2005). Understanding the impact HLEs have on language development can help caregivers and practitioners promote a language-rich environment. These results suggest that professionals working in early intervention settings should teach caregivers of toddlers with DS practical ways to embed literacy-related activities into their child’s everyday life as well as how to promote child engagement in shared storybook reading activities (e.g., holding the book, turning pages). Additionally, professionals could work with caregivers to teach what types of literacy materials are developmentally appropriate for their child and how to incorporate interactive reading strategies into shared storybook reading times to promote a literacy-rich environment. Lastly, all early childhood practitioners should actively involve caregivers in home treatment sessions and carryover programs to empower them to help their child acquire stronger communication skills.

## Data availability statement

The datasets presented in this article are not readily available because they include audio files that cannot be distributed. Requests to access the datasets should be directed to LM, ljhahn@illinois.edu.

## Ethics statement

The studies involving human participants were reviewed and approved by the University of Illinois at Urbana-Champaign IRB. Written informed consent to participate in this study was provided by the participants’ legal guardian/next of kin.

## Author contributions

MD, SL, and LM conceptualized the study and drafted and edited the manuscript. MD transcribed and coded the data. MD, SL, and LM assisted with data analysis. All authors contributed to the article and approved the submitted version.

## Funding

We received funding for this research study through the University of Illinois Campus Research Board Grant (PI: Mattie) and the Center for Health, Aging, and Disability Pilot Grant Program (PI: Mattie) from the University of Illinois.

## Conflict of interest

The authors declare that the research was conducted in the absence of any commercial or financial relationships that could be construed as a potential conflict of interest.

## Publisher’s note

All claims expressed in this article are solely those of the authors and do not necessarily represent those of their affiliated organizations, or those of the publisher, the editors and the reviewers. Any product that may be evaluated in this article, or claim that may be made by its manufacturer, is not guaranteed or endorsed by the publisher.
