# Supplementary material for: Home-literacy environments and language development in toddlers with Down syndrome
Source: Front Psychol. 2023 Jun 29;14:1143369. doi: 10.3389/fpsyg.2023.1143369 (PMC10340519; doi:10.3389/fpsyg.2023.1143369)
Supplement: Supplementary file 1 [file Data_Sheet_1.pdf]

Supplementary Material

Home Literacy Environments and Language Development in Toddlers with Down Syndrome

Madison S. Dulin*, Susan J. Loveall, & Laura J. Mattie

*** Correspondence:** Madison S. Dulin: madisondulin19@gmail.com

Home Literacy Environment (HLE) Questionnaire

Instructions: Please circle or write in your answers. If a question does not apply, please write "N/A" for not applicable.

1. Approximately how many children's storybooks do you currently have in your home?

__________ books

2. Approximately how many adult- level books do you currently have in your home?

__________ books

3. During a typical week, how many times do *you, yourself*:

a.) *Read books for enjoyment?*

Never 1- 2 times 3- 4 times 5- 6 times 7- 8 times 9- 10 times 11+

b.) *Read informative books (e.g. for work and/ or school)?*

Never 1- 2 times 3- 4 times 5- 6 times 7- 8 times 9- 10 times 11+

4. How much do you agree with the following statement?

"I enjoy reading storybooks with my child."

1	2	3	4
Strongly Disagree	Disagree	Agree	Strongly Agree

5. Please estimate, in months, the age of your child when you began reading to them.
__________ mo.

6. Do you have a designated reading time with your child? Yes No

If yes, when do you typically read with your child? Morning Afternoon Evening

7. Think about the past week. How many times during the week did you read to your child (including books, magazines, stories on e-readers, comic books, etc.)?

Never 1- 2 times 3- 4 times 5- 6 times 7- 8 times 9-10 times 11+

8. Think about the past week. How many books did you read to your child in the past week?

0 1- 2 3- 4 5- 6 7- 8 9- 10 11+

9. On average, how many books do you typically read to your child in one sitting?

N/A 1- 2 3- 4 5- 6 7- 8 9- 10 11+

10. Taking into consideration the length of the children's books you read together, on average, how much time do you read to your child in one sitting?

< 10 mins. 10- 20 mins. 21- 30 mins. 31- 40 mins. 41- 50 mins. 51- 60 mins. >1 hr.

11. Taking into consideration the length of the children's books you read together, on average, how much time per week do you read to your child?

< 15 mins. 15- 30 mins. 30- 45 mins. 1- 2 hrs. 3- 4 hrs. 5- 6 hrs. 7+ hrs.

12. How much do you agree with each of the following statements?

"During storybook reading time with my child, I regularly:

a.) *Point out details from the story (e.g., character's behaviors and emotions, point and name objects in the illustrations) that are outside the actual text."*

1	2	3	4
Strongly Disagree	Disagree	Agree	Strongly Agree

b.) *Relate what is happening in the story to my child's everyday interactions (e.g., bath time, bedtime, family relations)."*

1	2	3	4
Strongly Disagree	Disagree	Agree	Strongly Agree

c.) *Ask my child questions about the story and follow-up with answers."*

1	2	3	4
Strongly Disagree	Disagree	Agree	Strongly Agree

d.) *Teach the names of the letters in the alphabet and/or alphabet sounds when reading."*

1	2	3	4
Strongly Disagree	Disagree	Agree	Strongly Agree

13. In comparison to other activities (e.g. bath time, play time, snack time, etc.), how would you rate your child's interest in storybook reading?

1	2	3	4
Least favorite activity			Most favorite activity

14. Please estimate how many times you visited the library and/or bookstore with your child in <u>the last year</u>?

Never Once Every 4- 6 months Every 2- 3 months Monthly Every 2-3 weeks Weekly

15. On average, how many hours per week does your child watch TV?

N/A < 1hr. 1- 2 hrs. 3- 4 hrs. 5- 6 hrs. 7- 8 hrs. 9- 10 hrs.

16. During a typical week, how many times does your child:

a.) *Ask you to read to him/her?*

Never 1- 2 times 3-4 times 5-6 times 7- 8 times 9- 10 times 11+

b.) *Pretend to read the story in a book (e.g., sitting with a book and producing speech that is similar to the actual story in the book)?*

Never 1- 2 times 3-4 times 5-6 times 7- 8 times 9- 10 times 11+

This questionnaire is going to parents of children across a wide age range. Some of these skills may not be age- appropriate for younger children. If your child is too young for one of these skills, please select "Never."

17. During storybook reading time with your child, does he/ she:

a.) *Grab for/ hold the book?*

Never Has but rarely Occasionally A few times per story Very frequently during story

b.) *Turn pages with or without your help?*

Never Has but rarely Occasionally A few times per story Very frequently during story

c.) *Independently point out pictures or words on the page?*

Never Has but rarely Occasionally A few times per story Very frequently during story

d.) *Name pictures that he/ she is familiar with?*

Never Has but rarely Occasionally A few times per story Very frequently during story

f.) *Ask questions about characters/events from the story?*

Never Has but rarely Occasionally A few times per story Very frequently during story

g.) *Fill in words or lines from the story (e.g., when reading a book he/she knows well, says the next line or word before you read it)?*

Never Has but rarely Occasionally A few times per story Very frequently during story

Figure 1

Scatterplots for each independent variable and Words Understood at Time 1.

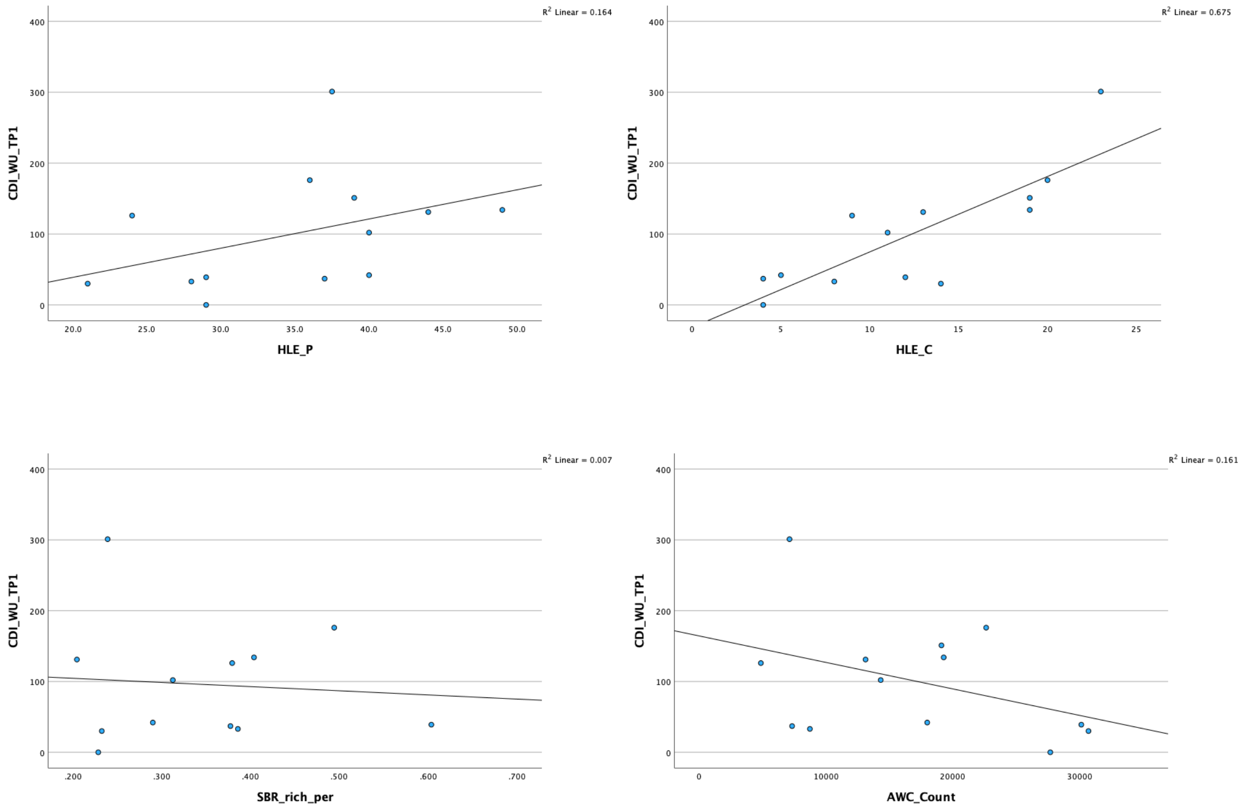

Note. CDI_WU_TP1 = words understood at Time 1, as measured by the CDI. HLE_P = richness of home literacy environment, as reported by the parent. HLE_C = Child engagement in shared book reading activities, as reported by the parent. SBR_rich_per = quality of a recorded shared book reading activity. AWC_Count = adult word count, as measured by the LENA.

Figure 2

Scatterplots for each independent variable and Words Understood at Time 2.

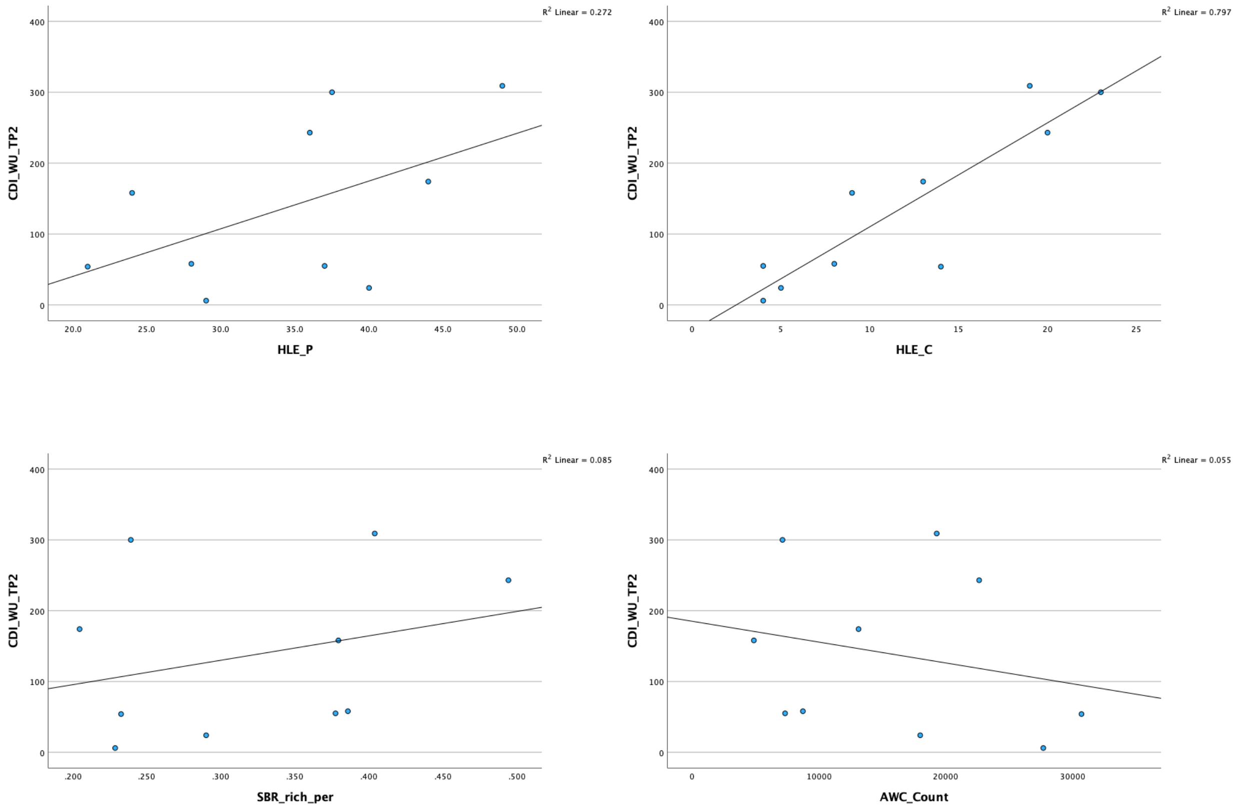

Note. CDI_WU_TP2 = words understood at Time 2, as measured by the CDI. HLE_P = richness of home literacy environment, as reported by the parent. HLE_C = Child engagement in shared book reading activities, as reported by the parent. SBR_rich_per = quality of a recorded shared book reading activity. AWC_Count = adult word count, as measured by the LENA.
